# Development of Halloysite Nanohybrids-Based Films: Enhancing Mechanical and Hydrophilic Properties for Wound Healing

**DOI:** 10.3390/pharmaceutics16101258

**Published:** 2024-09-27

**Authors:** Francisco Ramón Rodríguez Pozo, Daiana Ianev, Tomás Martínez Rodríguez, José L. Arias, Fátima Linares, Carlos Miguel Gutiérrez Ariza, Caterina Valentino, Francisco Arrebola Vargas, Pablo Hernández Benavides, José Manuel Paredes, María del Mar Medina Pérez, Silvia Rossi, Giuseppina Sandri, Carola Aguzzi

**Affiliations:** 1 Department of Pharmacy and Pharmaceutical Technology, Campus Cartuja s/n, 18011 Granada, Spain; frodriguezpozo@correo.ugr.es (F.R.R.P.); pabloj@ugr.es (P.H.B.); mdelmar@ugr.es (M.d.M.M.P.); carola@ugr.es (C.A.); 2 Department of Drug Science, University of Pavia, Via Taramelli 12, 27100 Pavia, Italy; daiana.ianev01@universitadipavia.it (D.I.); caterina.valentino01@universitadipavia.it (C.V.); silvia.rossi@unipv.it (S.R.); g.sandri@unipv.it (G.S.); 3 Institute of Biopathology and Regenerative Medicine (IBIMER), Center of Biomedical Research, University of Granada, 18016 Granada, Spain; 4 Biosanitary Research Institute of Granada (ibs. Granada), Andalusian Health Service (SAS), University of Granada, 18012 Granada, Spain; 5 Unit of Force Atomic Microscopy, Scientific Instrumentation Center, University of Granada, 18003 Granada, Spain; flinaor@ugr.es (F.L.); carlos.gutari@ugr.es (C.M.G.A.); 6 Department of Histology, Institute of Neurosciences, Centre for Biomedical Research (CIBM), University of Granada, 18071 Granada, Spain; fav@ugr.es; 7 Nanoscopy-UGR Laboratory, Department of Physical Chemistry, Unidad de Excelencia en Química Aplicada a Biomedicina y Medioambiente UEQ, University of Granada, Cartuja Campus, 18071 Granada, Spain; jmparedes@ugr.es

**Keywords:** atomic force microscopy, chitosan, films, halloysite, hydrophilic/hydrophobic character, hydrolyzed collagen, mechanical properties, nanohybrids, wound healing

## Abstract

Most of the therapeutic systems developed for managing chronic skin wounds lack adequate mechanical and hydration properties, primarily because they rely on a single component. This study addresses this issue by combining organic and inorganic materials to obtain hybrid films with enhanced mechanical behavior, adhesion, and fluid absorption properties. To that aim, chitosan/hydrolyzed collagen blends were mixed with halloysite/antimicrobial nanohybrids at 10% and 20% (*w*/*w*) using glycerin or glycerin/polyethylene glycol-1500 as plasticizers. The films were characterized through the use of Fourier-transform infrared (FTIR) spectroscopy, thermogravimetric analysis (TGA), and electron microscopy. The mechanical properties were evaluated macroscopically using tensile tests, and at a nanoscale through atomic force microscopy (AFM) and nanoindentation. Thermodynamic studies were conducted to assess their hydrophilic or hydrophobic character. Additionally, in vitro cytocompatibility tests were performed on human keratinocytes. Results from FTIR, TGA, AFM and electron microscopy confirmed the hybrid nature of the films. Both tensile tests and nanomechanical measurements postulated that the nanohybrids improved the films’ toughness and adhesion and optimized the nanoindentation properties. All nanohybrid-loaded films were hydrophilic and non-cytotoxic, showcasing their potential for skin wound applications given their enhanced performance at the macro- and nanoscale.

## 1. Introduction

Wounds trigger a physiological healing process that involves four partially overlapping stages: hemostasis, inflammation, proliferation, and remodeling [1,2]. Wounds remaining in the inflammatory stage for more than three months are classified as chronic wounds [3]. These injuries have a considerable economic impact on healthcare systems and compromise the quality of life of patients [4,5].

Traditionally, primary treatments for chronic wounds have involved cleansing and debridement. Despite both being crucial in the healing process, they are costly and have a low success rate (<50%). This is associated with frequent bandage and gauze changes, which can be painful for patients [6]. These procedures increase the risk of wound infections that require antibiotics, which could facilitate the development of antimicrobial resistance [7,8,9]. Indeed, chronic wounds are more susceptible to contamination, especially by *Staphylococcus aureus* and *Pseudomonas aeruginosa*, which are highly prone to form biofilms. These biofilms hinder wound healing, given their resistance to antibiotics, and promote an immune response that can lead to chronic inflammation. To address these challenges, various strategies have been employed to accelerate wound repair and regeneration, mainly through the local delivery of antimicrobials and growth factors using organic or inorganic biomaterials [10,11,12,13,14,15,16,17,18]. In particular, the development of hybrid bio-nanomaterials based on natural inorganic carriers (halloysite and bentonite) and antimicrobial non-antibiotic agents, such as chlorhexidine dihydrochloride (CHX) [19] and human lactoferrin (h-LF) [20], has been recently proposed. These nanohybrids (NHs), especially those based on halloysite (HAL) nanotubes, showed high cytocompatibility with human fibroblast cultures and improved in vitro biocidal effects. The high specific surface area and surface electrical charges of inorganic carriers may promote the interaction with bacterial membranes. Compared to synthetic nanocarriers HAL nanotubes are eco-friendly and cost-effective; thus, they are suitable for biomedical applications [21,22,23,24,25,26,27].

Actually, numerous research efforts are devoted to the formulation of biomimetic supports, which provide a regenerative substrate that allows for cell adhesion and proliferation and, thus, the regeneration of damaged tissues. In this scenario, those proposals based on biomolecules are preferred over synthetic compounds due to their low immunogenicity and high cytocompatibility [28]. Concretely, proteins and polysaccharides are investigated given their abundance, cost-effectiveness, and suitability for providing a native cell-like environment that may accelerate wound healing [29,30,31,32]. Generally speaking, the majority of the developed systems are mono-component, often resulting in inadequate moisture retention for optimal wound healing and poor mechanical properties, making the substrates brittle and non-adherent [33]. Combining materials with complementary properties could be a key strategy when engineering novel hybrid multifunctional products with enhanced mechanical, adhesive, and water absorption performance [34,35]. In this line, the combination of inorganic nanostructures and organic matrices could offer excellent opportunities to achieve major advances in biomedicine. 

Given these premises, in this work, HAL-CHX and HAL-hLF NHs were incorporated into chitosan (CS)/hydrolyzed collagen (HC)-based films. CS is characterized by hemostatic and fibroblast stimulation activities, and antimicrobial, antioxidant and anti-inflammatory properties [33,36,37,38]. HC can stimulate chemotactic fibroblast migration, as well as fibroblast and keratinocyte proliferation [39]. In addition, HC is a cost-effective and water-soluble biomolecule with improved organoleptic properties compared to native collagen [40]. Incorporating NHs into the films aimed to overcome the limitations of pharmacological monotherapies in treating these lesions and to reduce drug doses. NHs could also promote synergistic antimicrobial activity thanks to the natural antibacterial mechanism associated with clay minerals [41]. Moreover, combining peptides and polysaccharides may overcome the limitations of mono-systems, generating hybrid materials with enhanced absorption capacities, mechanical properties, and cell adhesion [42]. Loading HAL-antimicrobial NHs may further enhance these properties, as clay minerals could act as reinforcing fillers in thin film samples [43,44,45,46,47,48,49,50].

The morphology and structure of the NH-loaded films were characterized using electron microscopy, FTIR spectroscopy, and thermal analysis. The mechanical properties of these films were investigated macroscopically via tensile testing and at the nanometric scale using AFM and nanoindentation. A comprehensive thermodynamic study helped to determine the surface thermodynamics of the films. Cytotoxicity tests on human keratinocyte cells were also carried out to define the biomedical applications of the NH-loaded films. 

## 2. Materials and Methods

### 2.1. Materials

Halloysite (HAL) nanoclay (Merck Life Science S.L.U., Madrid, Spain) is an aluminosilicate (H_4_Al_2_O_9_Si_2_ × 2 H_2_O; 294.19 g/mol) consisting of hollow tubular particles (length in the range of 120 to 1500 nm, average value ≈ 360 nm; inner diameter ≈ 18 nm and external diameter ≈ 65 nm) [20]. 

Medium-molecular-weight chitosan (CS), polyethyleneglycol-1500 (PEG 1500), chlorhexidine dihydrochloride (CHX), recombinant human lactoferrin (hLF, expressed in rice and iron saturated), sorbitan trioleate (Span^®^ 85), Dulbecco’s Modified Eagle’s Medium–high glucose (4.5 g/mL) (DMEM), and heat-inactivated Fetal Bovine Serum (FBS) were purchased from Merck Life Science S.L.U. (Madrid, Spain). Bovine hydrolyzed collagen (HC) was obtained from Kelisema S.R.L. [Tavernerio (CO), Italy]. Glycerin (gly) was acquired from Fagron Ibérica S.A.U. (Barcelona, Spain). Thiazolyl Blue Tetrazolium Bromide [3-(4,5-Dimethyl-2-Thiazyl)-2,5-Diphenyl-2H-Tetrazolium Bromide, MTT] was procured from PanReac Applichem ITW Reagents (Barcelona, Spain). All of the other chemicals and solvents were of high-quality analytical grade and were used as procured.

### 2.2. Methods

#### 2.2.1. Preparation of the Nanohybrids (NHs)

The NHs were prepared by spray drying 100 mL of HAL/drug dispersions [19,20]. CHX (0.01% *w*/*v*) and hLF (5% *w*/*v*) drug solutions were prepared in distilled water and in pH 7.4 phosphate-buffered saline solution (PBS), respectively. HAL aqueous dispersions (1% *w*/*v*) were prepared by dispersing HAL in water at 23,000 rpm for 15 min (Ultra-Turrax^®^ T25 S5, IKA^®^—Werke GmbH & Co. KG, Staufen, Germany). Then, 50 mL of the HAL dispersion was added to 50 mL of CHX or hLF solutions. The resulting HAL/drug dispersions were spray dried (BÜCHI^®^ B-290, Massó Analítica S.A., El Prat de Llobregat, Barcelona, Spain) under experimental conditions: 0.7 mm nozzle, an airflow rate 439 L/h, an aspirator 40 m^3^/h (100%), a 9 mL/min feed rate, a nozzle air pressure of 6 bar, an inlet temperature of 200 °C, and an outlet temperature of 130 to 135 °C. The resulting NHs, hereafter referred to as HAL-CHX and HAL-hLF, respectively, were sieved under 45 μm and stored in a desiccator until use.

#### 2.2.2. Preparation of CS/HC Films and Their Loading with NHs

CS/HC-based films were prepared following a procedure modified from Ref. [51]: 50 mL of a 2% (*w*/*v*) CS solution was prepared by dissolving CS in a 1% (*v*/*v*) aqueous acetic acid solution, adjusted to pH 4.25 with a weak non-irritant base (triethanolamine), under continuous stirring during 12 h. Adjusting the pH of the acetic acid solution assured the complete dissolution of the polysaccharide during film preparation and enhanced the film’s integrity when in contact with an aqueous medium during application. HC was dissolved at a 4% (*w*/*v*) concentration in 50 mL of distilled water, in which Span^®^ 85 (0.1% *w*/*v*) had been previously dispersed (at 60 to 80 °C under magnetic stirring). The surfactant reduced foam formation during NH incorporation. The volume of water evaporated during heating was renewed once room temperature was reached and HC was added under stirring. Then, the CS and HC solutions were mixed in a 1:1 *v*/*v* ratio, to final concentrations of 1% (*w*/*v*) CS and 2% (*w*/*v*) HC. The plasticizers—gly for Group 1 films and gly/PEG 1500 for Group 2 films—were slowly added to the blends. The NHs (in equal parts HAL-CHX and HAL-hLF) were also added under stirring at 10 and 20% *w*/*w* relative to the total mass of CS and HC in the blends. Films without NHs were also prepared: F1 (containing gly) and F2 (containing gly/PEG 1500) (Table 1). 

The mixtures were then kept under magnetic stirring for 4 h, poured into 5 cm diameter silicone molds and dried at room temperature under a fume hood with an air flow of 0.4 m/s.

#### 2.2.3. Thickness and Weight Measurements

The thickness of the films was measured using a digital caliper with a measuring range of 0 to 150 mm and a resolution of 0.01 mm (Digital caliper CD-15DAX, Mitutoyo Corporation, Kawasaki, Japan). The thickness was determined by using five different measuring points and calculating the average values. After removal from the mold, weight measurements of the films were carried out using an analytical balance with an accuracy of ±0.1 mg (Boeco BBI-31 balance, Boeckel & Co. GmbH & Co. KG, Hamburg, Germany). 

#### 2.2.4. Mechanical Properties

Assessing the macroscopic mechanical properties of films, e.g., tensile strength (TS) and elongation at break (EB), is essential to ensure that they develop effective and consistent protection for chronic wound healing. They were analyzed using a Texture Analyser TX.AT plus 100 equipped with a 5 Kg load cell and a film support rig (HDP/FSR) (Stable Micro Systems, ANAME, Madrid, Spain). HDP/FSR provided a biaxial extension approach to the measurement of burst strength of film-like samples. The films were cut into 3 cm × 3 cm squares and sandwiched between a Perspex^®^ base and a holder with a circular opening. A semispherical probe was then pushed through this opening to perform extension and elasticity measurements. During the test, the maximum force to rupture the sample was recorded. The lifting speed of the probe was kept constant at 0.5 mm/s and the final distance between the probes was set at 10 cm. Mechanical properties, such as tensile strength (TS) and elongation at break (EB), were determined.

TS was calculated using Equation (1):

(1) $$\mathrm{TS}\;\left(\mathrm{MPa}\right)=\frac{F_{\max}}{A}$$

where F_max_ is the maximum force applied (N), and A is the resulting contact area between the probe and the sample (mm^2^). 

EB was calculated according to Equation (2), as described by [52]:

(2) $$\mathrm{EB}\;\left(\%\right)=\frac{L-L_{0}}{L_{0}}\;\times \;100$$

where L is the distance covered by the probe up to the break of film samples (in mm), and L_0_ is the initial distance between the probes (in mm). 

Tensile tests were carried out in quintuplicate in each film formulation.

#### 2.2.5. Scanning Electron Microscopy (SEM) and Energy Dispersion Spectroscopy (EDS)

SEM images were obtained with a Dual Beam AMBER X high-performance focused plasma ion beam-scanning electron microscope (FIB-SEM) (Tescan Group a. s., Brno—Kohoutovice, Czech Republic) equipped with a Schottky field emission electron source (hot cathode). Samples were sputter-coated with carbon by means of a sputter-coating Nanotech Polaron-SEMPREP2 (Polaron Equipment Ltd., Watford, UK) before they were examined. EDS was carried out using a ULTIM^®^ MAX 100 microanalyser (Oxford Instruments, High Wycombe Buckinghamshire, UK) with a retractable SDD detector, large active detection surface (100 mm^2^), energy resolution at 5.9 keV and software AZtec^®^ 5.0 SP1 (Oxford Instruments, High Wycombe Buckinghamshire, UK). 

#### 2.2.6. Atomic Force Microscopy (AFM)

Topographic measurements and imaging: AFM imaging was carried out in TAPPING mode in air using an NX-20 instrument (Park Systems, Suwon, Korea) and ACTA cantilevers (spring force constant K = 40 N m^−1^ and f = 320 kHz). Images were acquired as 256 × 256 pixels (scan rate 0.5–0.7 Hz) and analyzed using XEI software 4.3 (Park Systems, Suwon, Korea). This resolution allows for the capture of necessary details and features for analysis or visualization [53,54,55]. Representative images of the samples were obtained by scanning at least 3 different locations on at least 3 different samples of the same nature. 

Force spectroscopy: The characterization of the nanomechanical properties of the films was carried out by using the gentle PinPoint mode to acquire reproducible and reliable topography, stiffness, adhesion and elastic module maps [56]. The procedure consisted of the following: (i) the XY scanner stopped during acquisition; (ii) the tip approached the surface, measured mechanical properties and retracted from the surface over a few ms (4 ms) to achieve an interaction force preset (10 to 20 nN; and (iii) recorded the approach height maintaining the Z distance.

Conductive AFM (c-AFM) was carried out at room temperature with the gentle PinPoint c-AFM mode, allowing for the best of both spatial resolution and current sensitivity by using a VECA amplifier whilst minimizing the lateral forces with optimized current measurement over different sample surfaces. The same three steps described above were followed. The force was held constant while the current was measured. Then, the tip was retracted and moved to the next pixel. This method requires ≈ 30 min to obtain highly reproducible images of 256 × 256 pixel and eliminates friction, which reduces tip–sample deterioration. CONTSCPt probes were used (with platinum overall coating); these worere 225 μm long and had a resonant frequency of ≈23 kHz and a spring constant of 0.2 N m^−1^. Electric contact was made by applying a drop of silver conducting paste to one corner of the chip and to the metallic chuck. The current was measured directly after the tip using a preamplifier with a gain of 10^9^ V/A (VECA). c-AFM imaging was carried out with different applied bias (+1 V, +3 V and +5 V). The imaged area was separated ≈ 5 mm from silver paste contact.

#### 2.2.7. Nanoindentation Measurements

They were conducted using a nanoindenter (Hysitron TI Premier, Bruker, Billerica, MA, USA) equipped with a Berkovich diamond tip. The instrument measured indenter displacement and normal force while the indenter moved vertically at a constant speed. Eight to sixteen indentations were performed for each sample. The same loading function was used for each indentation: during the loading step, the load increased from 0 to 1200 µN over 20 s at a rate of 60 µN/s, and the maximum load was maintained for 30 s; in the unloading step, the load decreased from 1200 to 0 µN in approximately 6.5 s at a rate of 185 µN/s. These conditions were chosen to account for the viscoelasticity of the samples, requiring rapid unloading and moderate load to avoid exceeding the elastic limit of the sample. Holding the maximum load for an extended time was intended to reduce the viscoelastic adhesion effects [57]. The Oliver–Pharr method was used to calculate the nanoindentation modulus and hardness [58]. 

#### 2.2.8. Structural Characterization

The FTIR spectra of the films and NHs were obtained using the JASCO 6200 spectrophotometer with SPECTRA MANAGER v2 software and a built-in attenuated total reflectance (ATR) accessory (JASCO Inc., Easton, MD, USA). Measurements were carried out at wavenumbers between 400 and 4000 cm^−1^ (resolution of 0.25 cm^−1^ and 84 scans).

TGA: Thermograms of each film and NH were obtained with a TGA/DSC device with an FRS5 sensor and a precision microbalance (±0.1 μg) (Mettler-Toledo GMBH, Cornellà del Lobregat, Barcelona, Spain). Samples of 20 or 40 mg (depending on organic or inorganic nature, respectively) were used for heating in an N_2_ atmosphere at a rate of 5 °C/min (thermal ramp from 35 to 960 °C).

#### 2.2.9. Surface Thermodynamics

A full surface thermodynamic analysis was carried out to assess the hydrophilic/hydrophobic character of the films. In each film, the total interfacial solid (*S*)/liquid (*L*) free energy (γ_SL_^TOT^) was calculated as the sum of two contributions, known as the non-polar Lifshitz–van der Waals (γ_SL_^LW^) component and the acid–base (γ_SL_^AB^) component. The latter, in turn, is related to the electron donor (γ^−^) and electron acceptor (γ^+^) characteristics of both the *S* and *L* materials [59,60]. Young’s equation, relating the *SL* contact angles (θ) with γ_SL_^TOT^, was used in the calculations [61]. The θ of three liquids (water, formamide, and diiodomethane) on the films, cut in regular shapes (3 cm × 1 cm), was determined at 25.0 ± 0.5 °C using a TL101-Auto1 Theta Lite Optical Tensiometer (LASING S.A., Madrid, Spain), equipped with a CMOS 1/2″ USB 3.0 digital camera with fixed zoom and OneAttension software 4.1 (LASING S.A., Madrid, Spain), following the sessile drop method described in the European Pharmacopeia.

#### 2.2.10. In Vitro Cytotoxicity Test/Assay

The cytocompatibility of the film extracts was assessed in immortalized human keratinocytes (HaCaT, 300493, CLS Cell Lines Service GmbH, Eppelheim, Germany) using a colorimetric assay to identify cellular metabolic activity (MTT). Film extracts were prepared according to ISO 10993–12:2012 standards [62]: the films were cut into 0.5 cm diameter circles, sterilized using UV irradiation, and incubated overnight in 3 mL of complete medium (CM) consisting of DMEM supplemented with 10% FBS.

The cells were grown in polystyrene flasks containing CM under standard conditions (37 °C and 5% CO_2_ atmosphere; HeraScientific, Madrid, Spain). When the cells reached 80 to 90% confluence, cytocompatibility was tested. The cells were seeded using a multichannel pipette into 96-well plates at a density of 10,000 cells/well, with CM, and incubated for 24 h at 37 °C in a 5% CO_2_ atmosphere. After 24 h, the CM was removed, and 100 µL of the film extracts was added to each well. The cells were then incubated for either 24 or 72 h under these standard conditions. At the end of each incubation time, the film extracts were removed, and 200 µL of DMEM without FBS was added to each well. Then, 50 µL of MTT solution was added per well, and the cells were incubated for 4 h under standard conditions. Following MTT incubation, the medium was removed, and 200 µL of DMSO and 25 µL of Sorensen’s buffer were added to each well. The plate was then shaken at 100 rpm (Rotamax 120, Heidolph, Germany) for 15 min. Finally, the optical density of each well was determined at 562 nm (Universal Microplate Reader ELx800, Bio-Tek Instruments Inc., Santa Clara, CA, USA). Negative controls (containing CM only) and positive controls (HaCaT cells in CM) were used in the study. All of the experiments were carried out in sextuplicate. 

#### 2.2.11. Statistical Analysis

One-way ANOVA and post hoc Scheffé test were performed using STATGRAPHICS Plus 5.1 software package for Windows (Statgraphics Technologies Inc., The Plains, VA, USA). Differences between the experimental data were considered to be statistically significant at *p* < 0.05.

## 3. Results and Discussion

### 3.1. Thickness and Weight Measurements

Table 2 shows that for each group of films, thickness increased with increasing solid content in their composition: from ≈128 to ≈229 μm (Group 1), and from ≈ 152 to ≈ 271 μm (Group 2). Film weight ranged from ≈375 to ≈428 mg in formulations containing gly (Group 1), and from ≈454 to ≈557 mg in formulations containing both plasticizers (Group 2). Hence, weights increased with increasing NH concentrations.

### 3.2. Mechanical Properties

The macroscopic mechanical behaviors of the films were investigated to evaluate the mechanical resistance (TS) and the toughness (EB) of the films (Table 3). In the treatment of chronic wounds, which requires long-term effectiveness, TS and EB are key characteristics in terms of ensuring that films can withstand stresses and strains without tearing or degrading. In fact, films must conform well to the skin and stay securely in place without causing discomfort. To that aim, toughness is a key factor in terms of their ability to adapt to body movements and contours. The results were obtained using the platform rig (HDP/FSR), which is specifically designed for film-like samples. It produces a more complete and realistic (biaxial) deformation compared to conventional probes, which only measure uniaxial vertical stretch (e.g., clamps). Samples were stretched horizontally while simultaneously coming into contact with the spherical probe that moved down vertically.

The TS values of the NH-loaded films decreased compared to both F1 and F2 films. It had been previously described how the TS values of organic films containing clay mineral particles as fillers decreased at inorganic phase concentrations of >5 to 7%. Such a reduction could be due to the accumulation of a high concentration of clay particles, reducing the stress-induced alignment of inorganic components in polymer matrices [44,45,47,63]. 

The EB values of the films loaded with 10% of NHs were significantly higher than those of the corresponding F1 and F2 films. The EB values did not change significantly at NH concentrations of 20%. It could be due to the plasticizing effect of NHs, which increased the ductility of the films. This plasticizing effect on polymeric films was previously described in HAL nanotubes loaded with antioxidant agents [43]. 

A decrease in both TS and EB was observed when comparing Group 1 and Group 2 formulations. It was previously described how the EB values increased as the gly content was increased, while they decreased as PEG 1500 concentrations increased. In addition, a significant interaction between the effects of both plasticizers was observed by using a full factorial experiment design [51]. These results suggested that the Group 1 films, i.e., F1-10, could be characterized by enhanced durability, flexibility, and protection in terms of managing chronic skin wounds. The higher mechanical resistance (TS) of the film favors the formation of a stable, protective layer, even against movements or pressures at the site of the wound. Furthermore, the greater toughness of the film may facilitate better conformation to the wound and surrounding tissue, minimizing detachments or damages during movement. These characteristics could increase patient comfort, particularly in areas where there is frequent movement, which can lead to better patient compliance and improved wound healing.

### 3.3. Scanning Electron Microscopy (SEM) and Energy Dispersion Spectroscopy (EDS)

**Figure 1:**
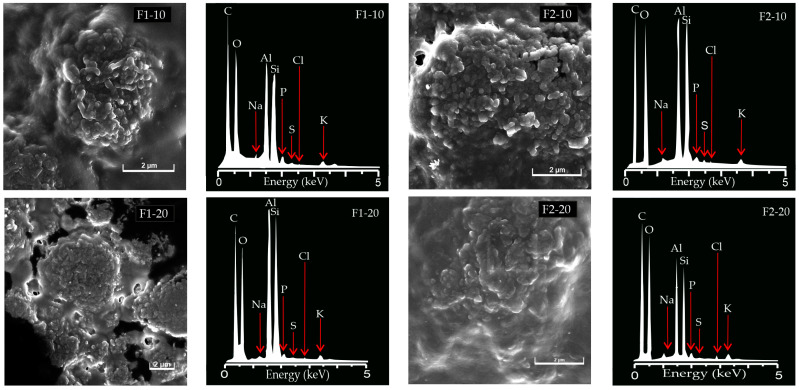
SEM microphotographs and EDS spectra of the NH-loaded films.

The morphology and surface chemistry of the NH-loaded films were evaluated through the use of SEM and EDS. These characteristics may determine the adhesiveness and hydrophilic/hydrophobic characteristics of the films. Abundant and equally distributed inorganic phases are shown in Figure 1. The aggregates of HAL nanotubes were observed, along with several pores and depressions. The loading of NHs to the films was also demonstrated using EDS: Si, O, Al, Na, S, Cl, K, and P peaks are characteristics of HAL-CHX and HAL-hLF nanohybrids [19,20]. The C peak could be ascribed to CS and HC and to sample preparation for SEM analysis. EDS microanalyses of the unloaded films were not possible because of their instability under electron beams.

### 3.4. Atomic Force Microscopy (AFM)

#### 3.4.1. Topographic Measurements and Imaging

Topographic determinations provided a detailed insight into the surface characteristics of the films. They allowed for a closer examination of surface nano-details and small-scale topographical variations, generating complementary data to SEM observations. Topographic analysis was essential to detect differences in the phase shift of the cantilever’s sinusoidal vibration signal, thanks to surface scanning in an intermittent contact mode (Tapping). The phase shift highlighted light and dark areas with different properties due to the variety of materials in the films.

**Figure 2:**
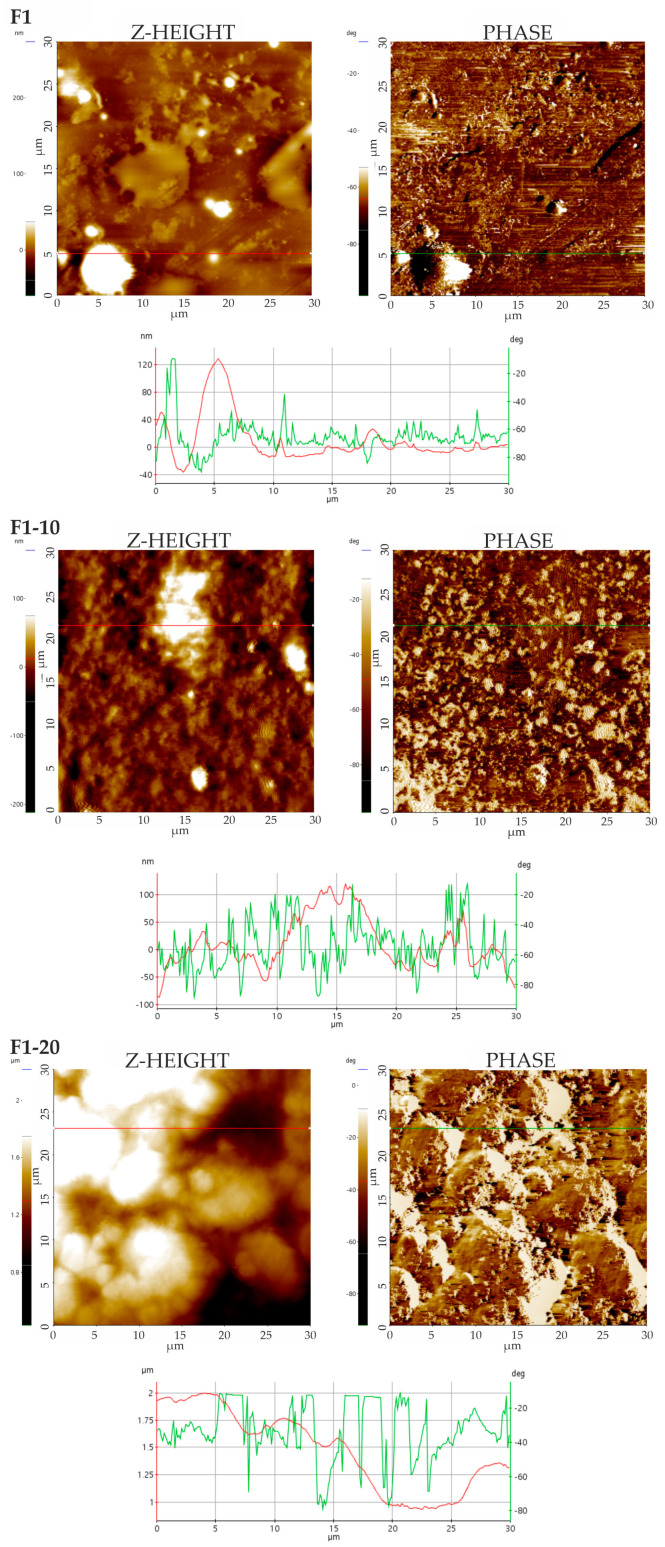
AFM images (TAPPING mode) of Group 1 films. Z-height (**left**) and phase images (**right**). Dual Line profiles from the Z-height image (red line) and phase image (green line) are also provided.

**Figure 3:**
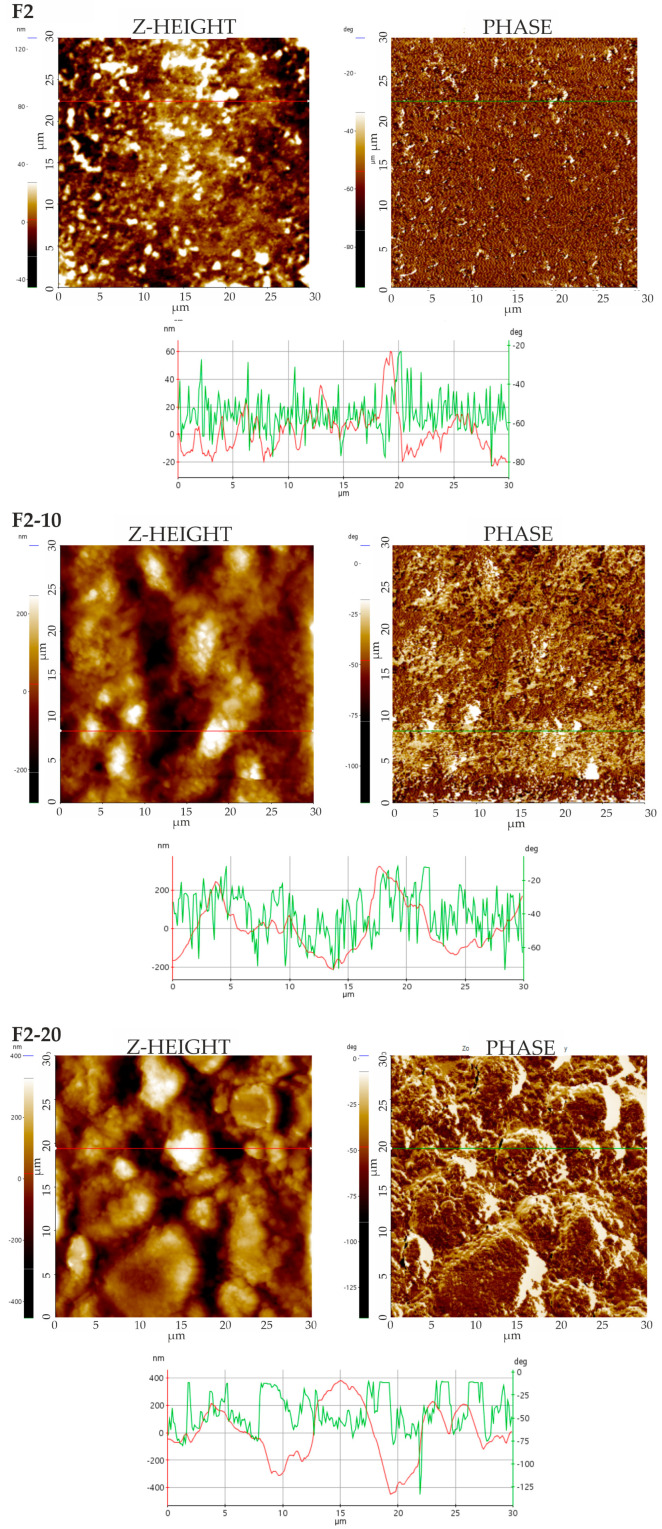
AFM images (TAPPING mode) of Group 2 films. Z-height (**left**) and phase images (**right**). Dual Line profiles from the Z-height image (red line) and phase image (green line) are also provided.

Representative AFM images (TAPPING mode) of F1, F2, and NH-loaded films are shown in Figure 2 and Figure 3. Marked differences were clear in the Z-height (topography) images, displayed as colored maps, with a color bar ascribing the color to Z-height. Concretely, the F1 films were characterized by Z-height values of <100 nm and isolated peaks exclusively between 100 and 200 nm. Contrary to this (see Figure 2), F1-10 and F1-20 films were characterized by greater Z-height values in the range of 100 to 200 nm or up to 2 μm, respectively. Increases in solid phase concentration, peaks (light areas), and valleys (dark areas) were observed on the surface. The increase in the Z-height values could be associated with an increase in NH concentrations on the film surface. In Group 2, even low NH concentrations provided a cardboard-like surface and greater Z-height values to the films (Figure 3). This increase in the surface was more evident in the F2-20 film, which was also characterized by greater Z-height values (400 nm). Phase images visualized the phase shift of the sinusoidal cantilever vibration signal, which was affected by the chemical composition of the samples. Images of the F1 and F2 films confirmed the heterogeneity of the samples, which were made by mixing various organic components, mainly CS and HC. A pattern of two phases with different contrasts was also observed in the NH-loaded films, thus confirming the hybrid nature of the formulations. In the films with high concentrations of NHs, i.e., F1-20 and F2-20, light phases prevailed over dark phases, probably due to the NH distributions on the sample surfaces. Alternatively, lighter phases could be ascribed to crystalline phases, while darker phases could be ascribed to phases rich in biomolecules [53].

#### 3.4.2. Force Spectroscopy

**Figure 4:**
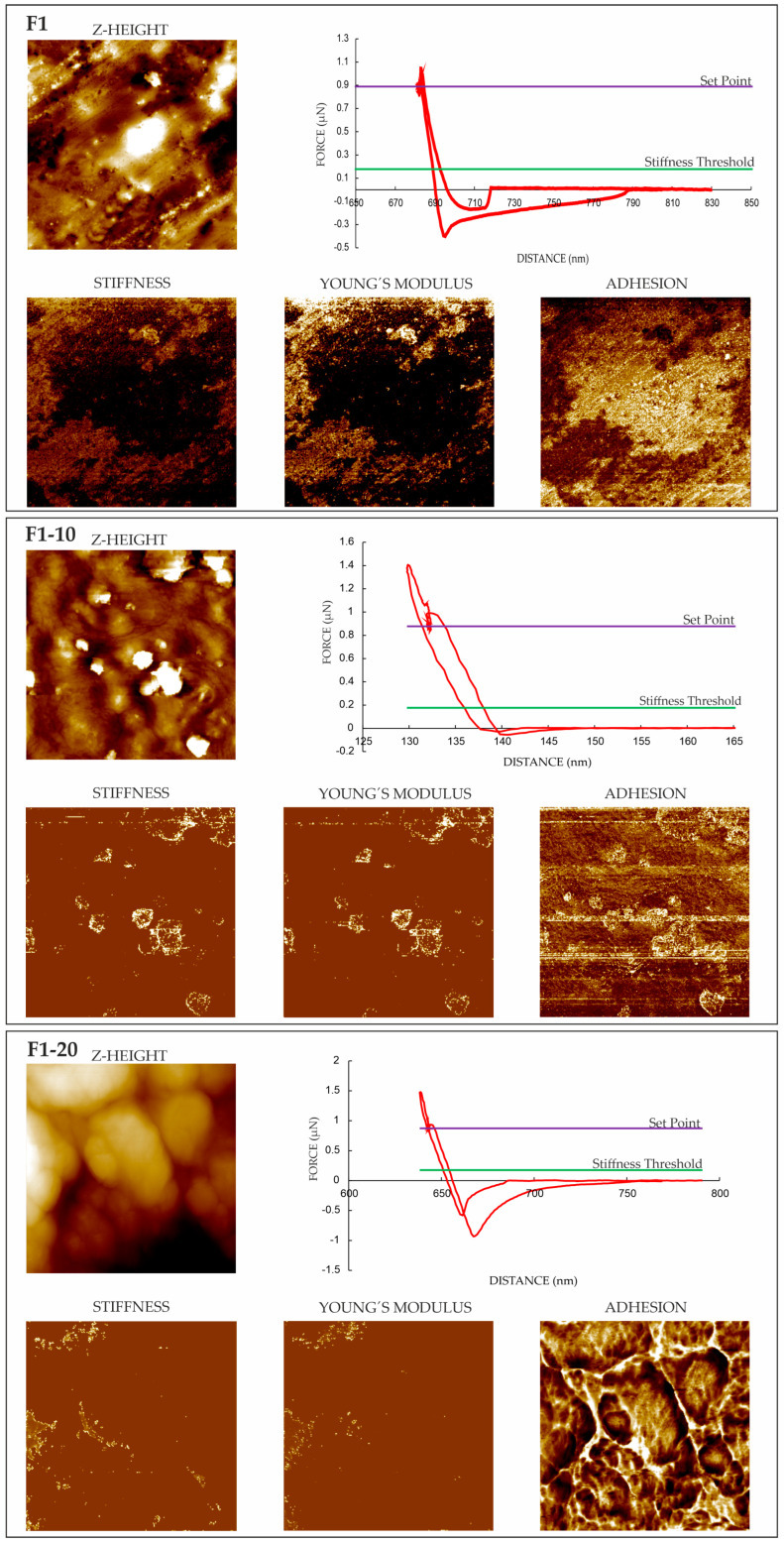
AFM Z-height images, F-D curves (red lines) and nanomechanical maps showing stiffness, Young’s modulus, and adhesion distribution of Group 1 films.

**Figure 5:**
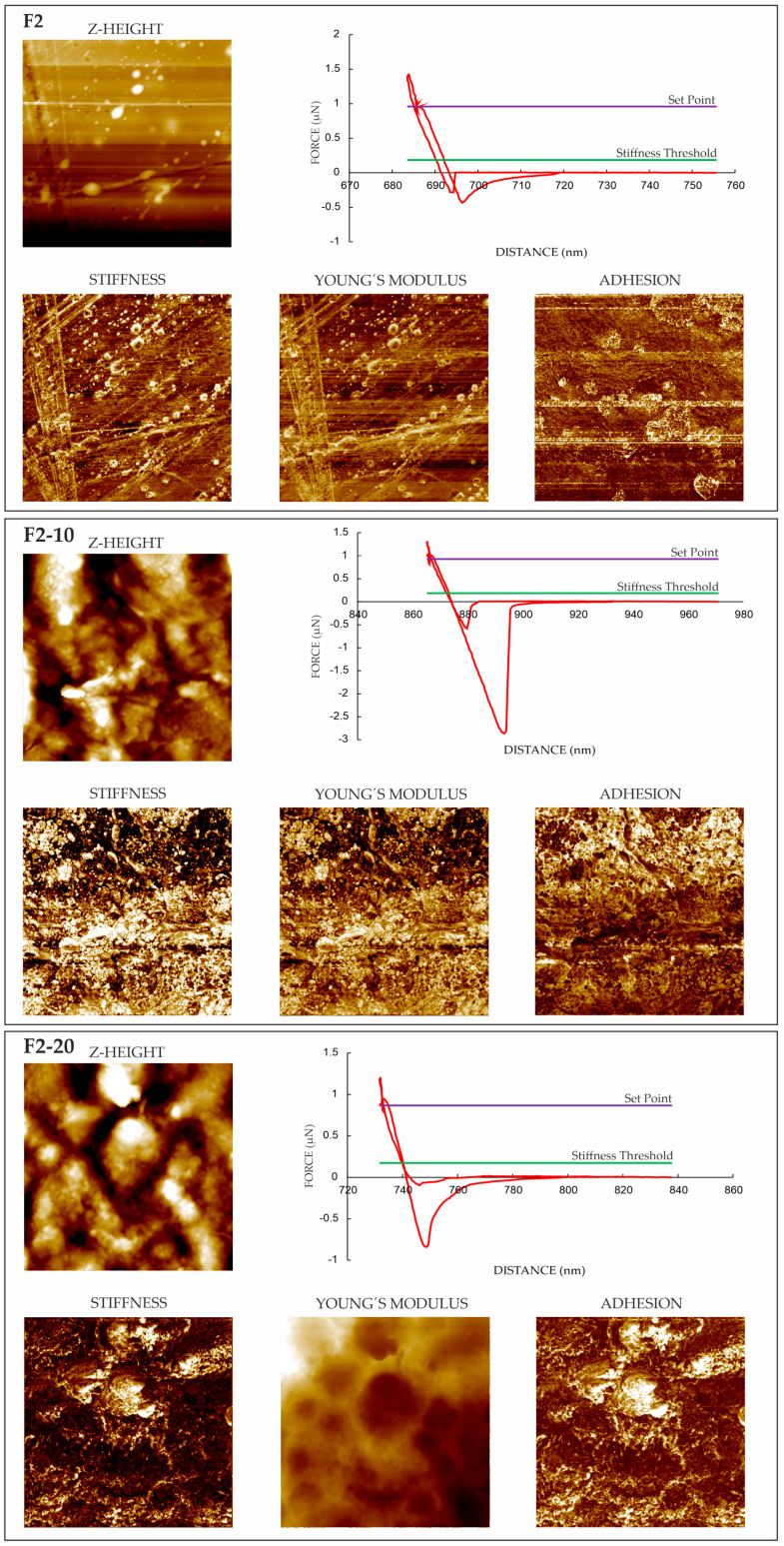
AFM Z-height images, F-D curves (red lines) and nanomechanical maps showing stiffness, Young’s modulus, and adhesion distribution of Group 2 films.

AFM also helped in terms of analyzing the nanomechanical properties of the films, e.g., stiffness, Young’s modulus, and adhesion forces. After identifying the phases in TAPPING mode (see Figure 2 and Figure 3), property mappings were conducted to define their distribution in the films. While macroscopic characterizations provided an overall understanding of the mechanical behavior of each film as a whole (see Section 3.2), AFM offered a powerful tool for exploring and understanding properties at the nanoscale, providing specific details on local variations existing within the samples. The nanomechanical properties extracted from each force–displacement (*F-D*) curve are compiled in Table 4. The values presented in this table are the averages of all the determinations conducted point by point at the nanoscale, which varied depending on the local phase distribution in the samples. This could justify the relatively high standard deviations obtained. Those points where the *F-D* curves were performed were selected manually over the phase image derived from the TAPPING mode, looking for different chemical and mechanical properties. The distribution maps of the properties of each film are plotted in Figure 4 and Figure 5, where light areas are associated with high values, while dark areas are related to low values for each property.

Similar stiffness was characterized for all films (Table 4). Comparable values of Young’s modulus were obtained for the Group 2 films, while a NH content of 10% in the Group 1 films increased the elastic modulus. Given that the experimental data from the AFM curves are basically qualitative, to clarify the behavior and accurately quantify the nanomechanical properties, the films were characterized using nanoindentation measurements (see Section 3.5). The inclusion of NHs in the films increased the adhesion forces of all the films compared to the F1 and F2 formulations. Adhesion on a nanoscale is measured by using the pull-off force when the tip of the needle is moved closer to the surface of the material and then suddenly repelled. As observed in the topographic measurements (tapping and phase images; see Figure 2 and Figure 3), the NHs were located in the superficial areas of the films. This NH distribution on the film surface could enhance the exposure of free silanol groups from HAL, thereby increasing the number of attractive sites on the films. The light areas shown in Figure 4 and Figure 5 correspond to the zones of high adhesion and could be associated with NHs in the films.

#### 3.4.3. Conductive AFM Measurements

**Figure 6:**
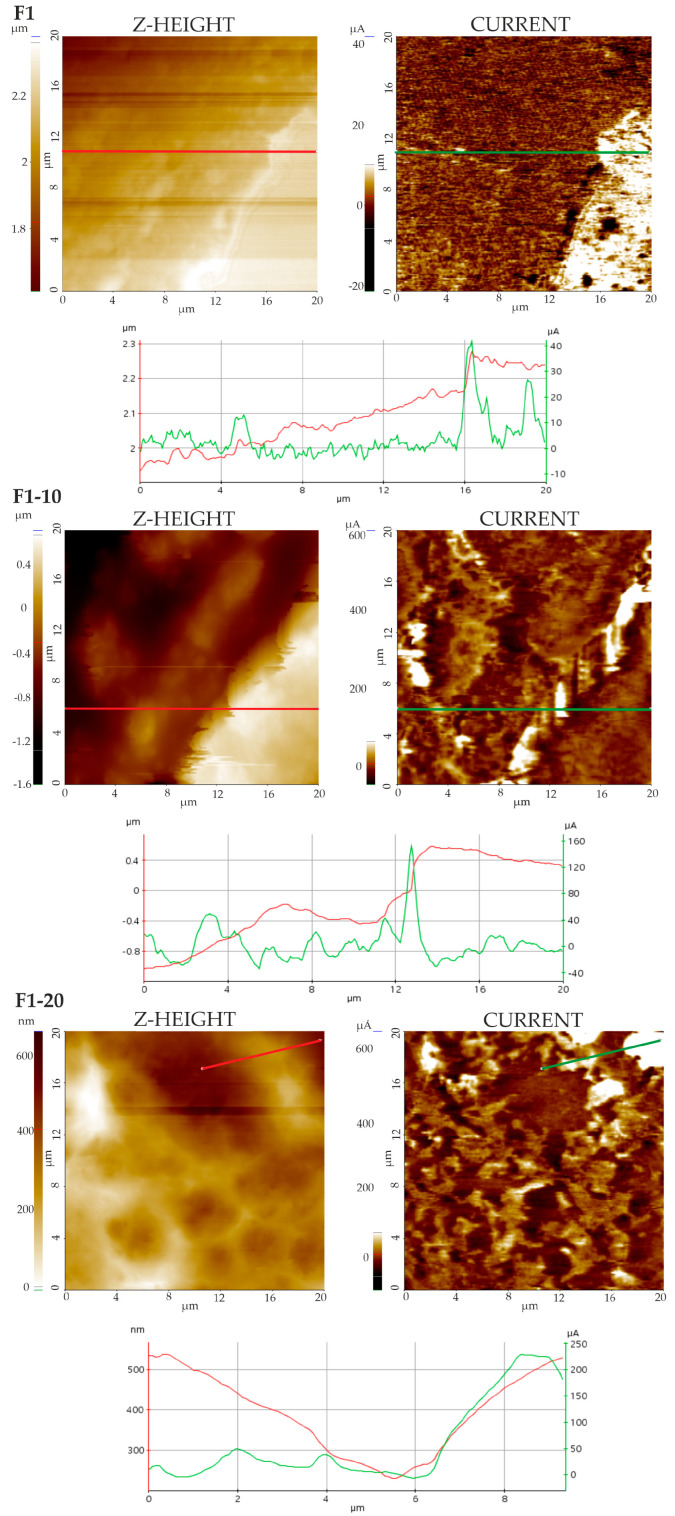
PinPoint c-AFM height image of Group 1 films, including the following: Z-height image; current signal at +1 V and Dual Line profiles from the Z-height image (red line) and current map (green line).

**Figure 7:**
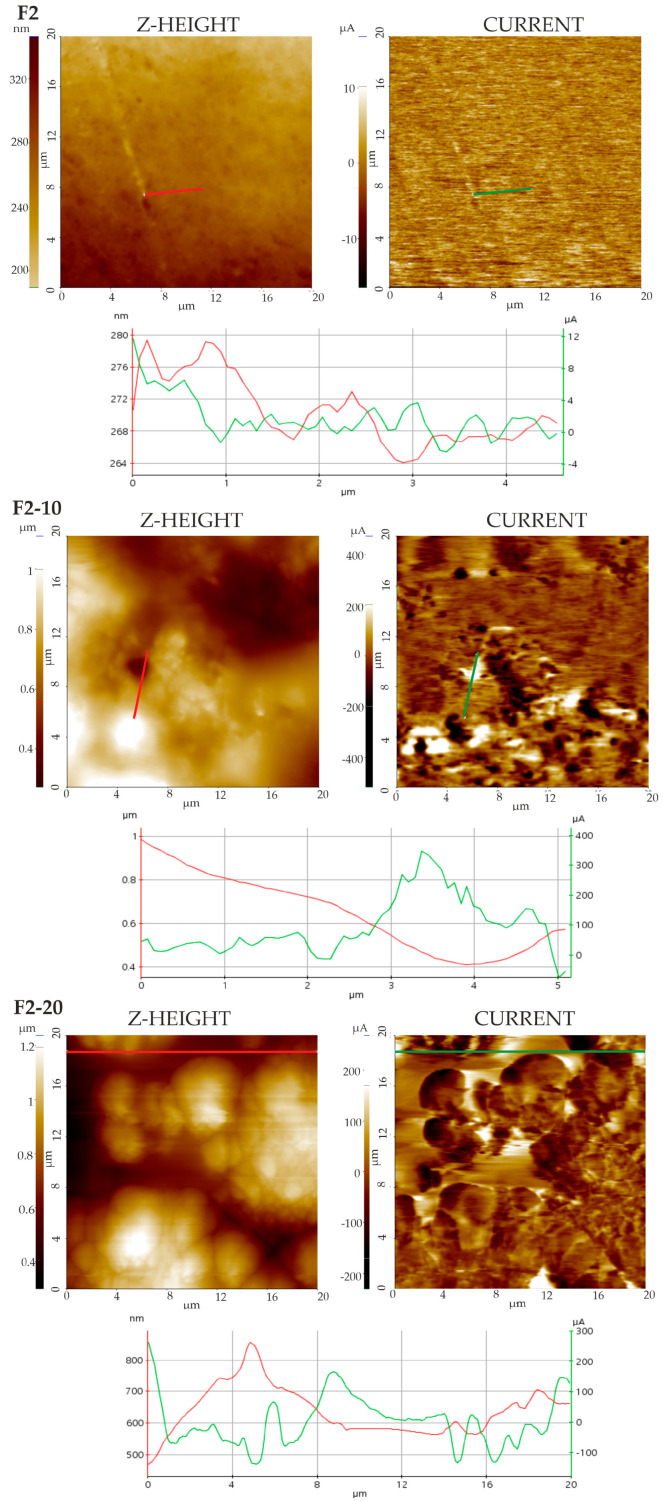
PinPoint c-AFM height image of Group 2 films, including the following: Z-height image; current signal at +1 V and Dual Line profiles from the Z-height image (red line) and current map (green line).

c-AFM measurements were carried out to check the hybrid nature of the films (NH loading), which is considered crucial in terms of assuring optimized mechanical and hydration properties. Determinations were carried out at voltages ranging from −3 to +3 V in each sample, and the signal became saturated in the presence of NHs. Hence, measurements taken at 1 V were selected (before saturation of the signal), and the results are shown in Figure 6 and Figure 7. F1 and F2 films displayed weak conductive properties in the range of 15 to 40 µA (F1) and 10 to 20 µA (F2). The conductive properties of the NH-loaded films increased to maximum values between 400 and 600 µA, even at 10% NH concentrations. Such an increase in conductance confirmed the incorporation of NHs into the film structures. Both NHs displayed surface charge properties, as defined previously: ζ-potential of approximately −8 mV and −20 mV, respectively [19,20]. In addition, the conductance distribution in the NH-loaded films was observed: light areas are associated with high conductivities (positive BIAS), and the dark areas correspond to low conductivities (negative BIAS). These observations confirmed the hybrid character of these formulations.

### 3.5. Nanoindentation Measurements

Nanoindentation measurements were carried out to quantitatively determine the nanomechanical properties of the films. The average values of hardness (H) and reduced elastic modulus (E_r_) are shown in Table 5. H and E_r_ values for Group 2 films, containing both gly and PEG 1500, were significantly lower than those for Group 1 formulations. Full factorial designs presented in previous works have revealed an interaction between the effects of both plasticizers on the mechanical properties (EB) of unloaded films [51]. Furthermore, higher H and E_r_ values were observed for NH-loaded films compared to the unloaded formulations, suggesting the reinforcing effect of NHs on the nanomechanical performance of the films. This behavior could controvert with the TS results from tensile tests. However, the extreme complexity of the formulations could complicate a clear definition of potential relationships at different scales. The correlations between mechanical properties at multiple scales are described in the literature, but with very well-defined materials, containing highly similar and homogeneous polymeric networks [64]. In addition, these differences could be attributed to the different characteristics evaluated in each test. Macroscopic tensile tests characterized the overall mechanical resistance of the films on a larger scale, where the presence of NHs could create stress concentrations at the film surfaces (as previously hypothesized), weakening their ability to withstand tensile forces. In contrast, nanoindentation evaluated the hardness at a nanoscale, focusing on localized regions of the materials. The increase in hardness observed through nanoindentation suggested that NHs enhance the material’s resistance to localized deformation or indentation. This could improve the film’s durability and resistance to wear and tear at specific points of contact, thereby enhancing protection and support in localized areas. This, in turn, might contribute to more effective management and healing of chronic wounds.

### 3.6. Structural Characterization

#### 3.6.1. Infrared Spectroscopy

**Figure 8:**
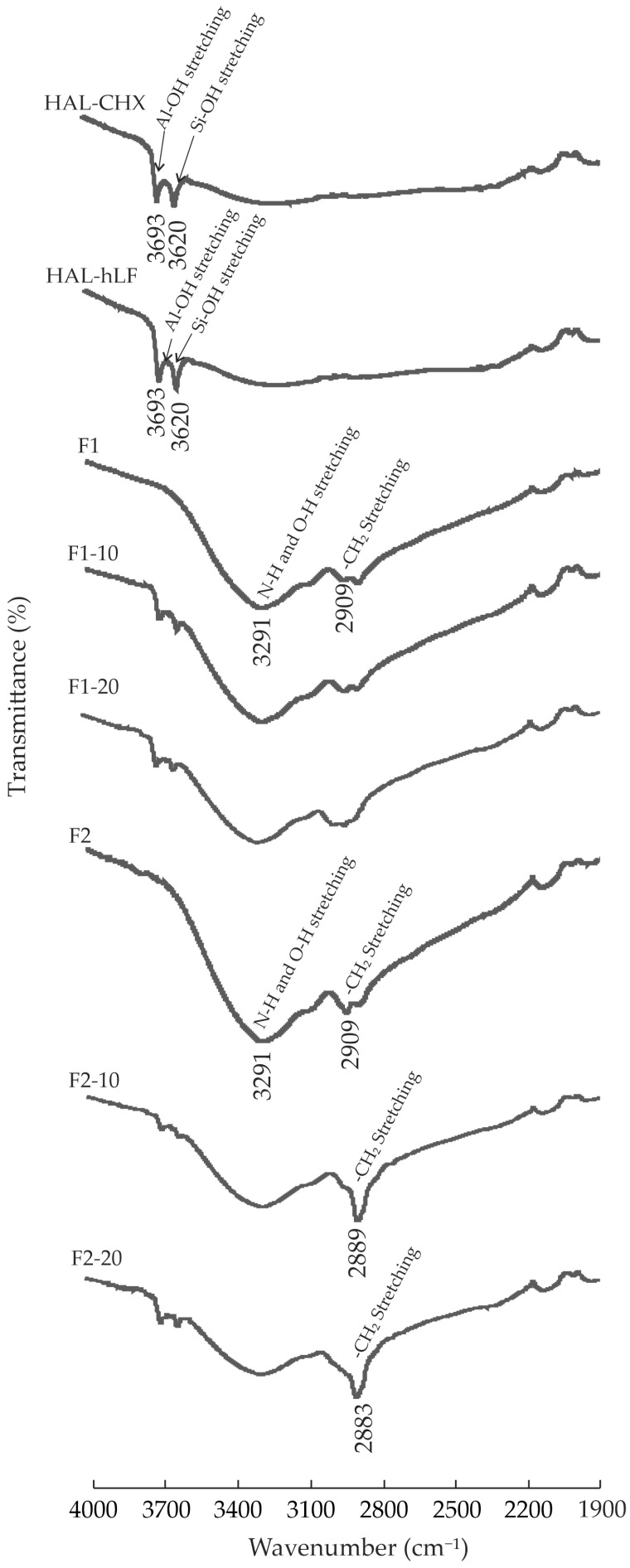
FTIR spectra of HAL-CHX and HAL-hLF NHs and films.

The ATR-FTIR spectra of the films are plotted in Figure 8. The spectra of the pristine components (CHX, hLF, CS, and HC) and NHs were studied in previous works [19,20,51]. All of the films displayed intense bands from overlapped N-H and O-H (3291 cm^−1^) and from CH_2_ stretching vibrations (2909 cm^−1^) of CS and HC. The intensification of these bands due to the incorporation of plasticizers, compared to pristine CS/HC films, was described previously [51]. However, IR peak shifts were not detected between the F1 and F2 films after the incorporation of the small mass of PEG 1500 (0.6 g), probably because of its uniform distribution in the formulations. Furthermore, the relatively similar chemistry of gly and PEG 1500 could determine that adding PEG did not introduce new chemical interactions or alter the environment of the functional groups to induce relevant shifts in the IR spectra. Varying the mass of PEG 1500 could bring light to this hypothesis, but it is out of the scope of this work.

The main bands of the NHs can be observed in the spectra of the loaded films, thus confirming the hybrid inorganic/organic nature of the formulations. Concretely, bands of hydroxyl stretching vibrations of HAL at 3693 cm^−1^ (inner Al-OH) and 3620 cm^−1^ (outer Si-OH) were identified. A broadening and slight intensification of the CH_2_ stretching band was also observed in F1-20, while in F2-10 and F2-20, this band was intensified and shifted slightly to lower values (2883 cm^−1^ in F2-20). These results could suggest hydrogen bonding interactions between the NHs and the CS and HC chains in the loaded films. Specifically, doubling the NH mass could increase the surface area available for hydrogen bonding, thus probably generating a greater number of hydrogen bonds in the resulting films. Hence, the F2-20 film, with greater NH content, might be characterized by a more extensive hydrogen-bonding network, resulting in greater shifts in the CH_2_ stretching wavenumber compared to the F1-10 films. Overlapping of the remaining principal bands of the components did not allow for the identification of more shifts in the wavenumbers of the vibrations coming from interactions between NH and organic components.

#### 3.6.2. Thermogravimetric Analysis (TGA)

**Figure 9:**
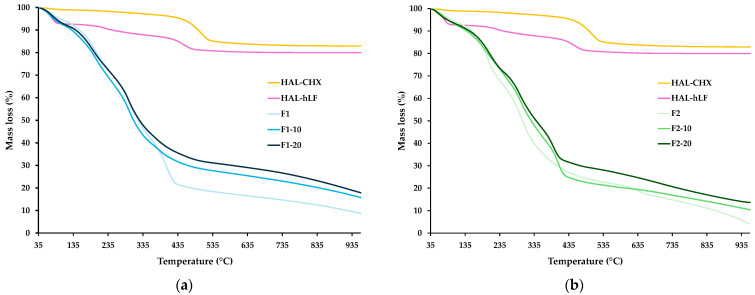
TGA curves of the films and NHs: (**a**) Group 1; (**b**) Group 2.

TGA measurements confirmed the hybrid nature of the films and defined their thermal stability and degradation behavior. This information is relevant to determining how the films will behave under different conditions, including environmental exposure, which is crucial to maintaining functionality in wound care. TGA curves of the films and NHs are plotted in Figure 9a,b. The thermal behaviors of the F1 and F2 films and NHs were consistent with the literature [19,20,51]. The mass loss in F1 and F2 curves was associated with the loss of superficial water and the degradation of acetic acid residues (at 35 to 200 °C). At higher temperatures, dihydroxylation, the loss of absorbed water molecules of HC, and the combustion of organic matter took place. NHs showed steps of residual moisture loss (at 35 to 100 °C), denaturation and/or degradation of the antimicrobial agents (>230 °C), and dehydroxylation of the aluminol groups of HAL (at 400 to 550 °C). Regarding the NH-loaded films, intermediate profiles were observed between the curves of the corresponding unloaded films and the NHs, which confirmed the existence of organic and inorganic phases in the films. Moreover, comparing the residual masses of the NH-loaded films and their corresponding unloaded formulations, thermal stability could be gained when the NHs are incorporated into the film structure. In fact, at the end of the heating (960 °C), NH-loaded Group 1 films had 15.7% (F1-10) and 17.8% (F1-20) mass residues vs. 8.6% of F1. A similar behavior was found for Group 2, where NH-loaded films showed residual masses of 10.4% (F2-10), and 13.7% (F2-20) vs. 4.1 (F2). The highest thermal stability was obtained at the greatest NH content (F1-20 and F2-20). The thermo-stabilizing effect of nanoclay particles in HAL/CS [45] and HAL/CS/PVA/PVP films has been previously hypothesized [48]. HAL nanotubes may limit the mobility of CS chains, and volatile degradation products must bypass the tortuosity offered by clay particles, thus delaying a mass transfer.

### 3.7. Surface Thermodynamics

The contact angle goniometry of the unloaded F1 and F2 films and NH-loaded films helped characterize their hydrophobic/hydrophilic characteristics. Similar studies were performed to define the surface thermodynamics of ethylcellulose-based [65] and CS-based [66] NHs with potential applications in biomedicine. It is known that the films used for wound management should exhibit adequate hydration properties to prove optimal hydration conditions at the wound site [67].

**Figure 10:**
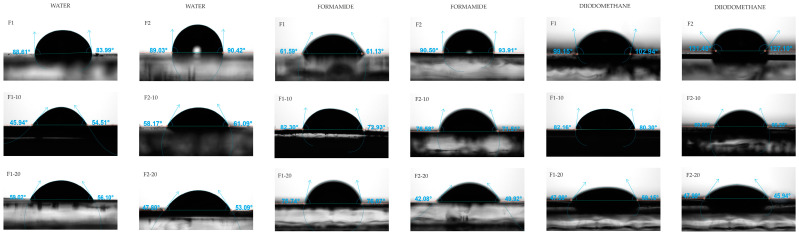
Representative images of the contact angles (θ) of water, formamide, and diiodomethane on the films.

In each formulation, contact angles (θ) of water, formamide and diiodomethane on the films were measured. Representative images are shown in Figure 10.

**Figure 11:**
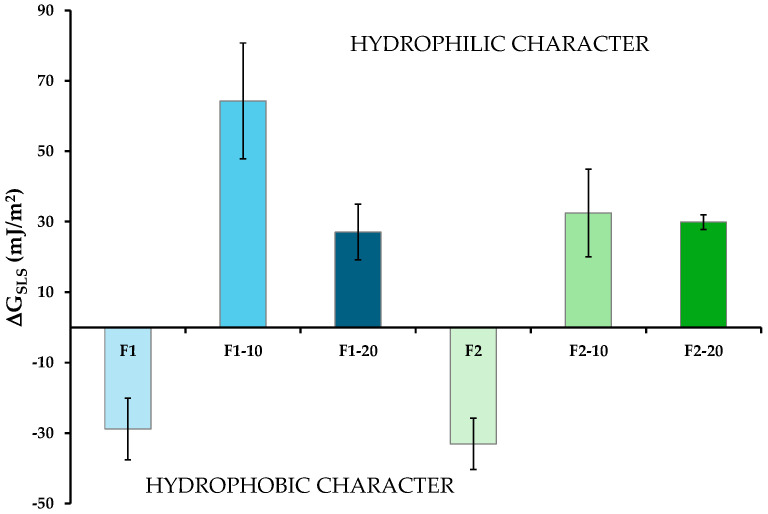
ΔG_SLS_ values of the films (mean values ± s.d; n = 6). One-way ANOVA, post hoc Scheffé test (*p* < 0.05: F1 vs. F1-10, F1-20; F1-10 vs. F1-20, F2-10; F2 vs. F2-10, F2-20).

Significant differences were observed among the films, particularly between the unloaded and NH-loaded formulations. The evaluation of the interfacial free energy components (γ) provided an accurate physical characterization of the surface thermodynamics of the formulations. This information is shown in Table 6. These values were used to calculate the surface free energy of interaction (∆G_SLS_) between the solid (films) and the three liquids (plotted in Figure 11), which defines the hydrophilic or hydrophobic characteristics of the samples [59]. Concretely, the greater the absolute value of ΔG_SLS_, the greater the hydrophilic or hydrophobic character of the film.

The results demonstrated that the addition of NHs to the films generated hydrophilic surfaces. As can be observed in Figure 11, the hydrophobic nature of F1 and F2 (negative ∆G_SLS_) changed to hydrophilic (positive ∆G_SLS_) in all the NH-loaded films. Regardless of the formulation group, the hydrophilic character did not increase significantly at the greatest NH concentrations. This behavior might be due to the higher presence of peaks in the films loaded with NHs, as observed in the topographical images obtained via AFM. The hydrophilic/hydrophobic properties of films depend, among other factors, on their texture. This characterization is consistent with previously published data [46], postulating that the θ of water in HAL/sulfated galactan bio-nanocomposites decreases as the clay mineral content increases (up to 15%). At high concentrations, the aggregation of inorganic nanoparticles may lead to an increase in surface roughness, limiting further increases in the hydrophilic characteristics of the formulation [68].

### 3.8. In Vitro Cytotoxicity Test/Assay

**Figure 12:**
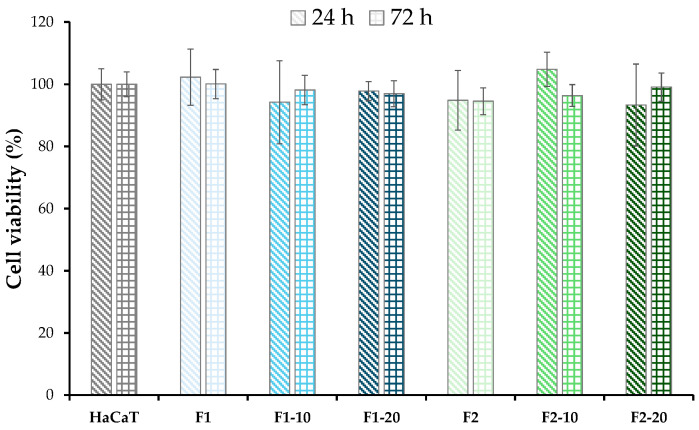
Relative cell viability (%) of HaCaT cells after 24 h and 72 h of incubation with the unloaded and NH-loaded films. HaCaT cells seeded in CM were considered a positive control (mean values ± s.d; n = 6).

Cytotoxicity measurements were carried out as proof of concept regarding the safety of these films in the treatment of skin wounds. Relative cell viability values (RCV, %) of HaCaT are plotted in Figure 12 after up to 72 h of incubation with the unloaded or the NH-loaded films. In all films, RCV was ≈100%, with negligible differences between 24 h and 72 h when compared to the positive control. These results suggested the lack of toxicity of the films with HaCaT cells. No significant differences were defined between NH-loaded films and unloaded films. This suggested that cell viability was not hindered by the NHs at any of the concentrations investigated. The cytocompatibility of the NHs was also demonstrated in human fibroblast cultures [19,20].

## 4. Conclusions

The differences between CS/HC-NH-loaded and CS/HC films have been described in terms of morphology, mechanical properties, structure, and surface thermodynamics. The hybrid nature of the films was confirmed via structural FTIR and TGA characterizations, revealing possible hydrogen bonds between NHs and organic networks, as well as improved thermal stability in NH-loaded films. Macroscopic mechanical properties showed that the incorporation of NHs into the films increased their toughness (particularly in formulation F1-10), which could favor their suitability for their application to wound beds. Descriptions of the nanomechanical behaviors of the films were detailed in maps depicting the distribution of stiffness, Young’s modulus, and adhesion forces within the films. NH-loaded films also showed enhanced adhesion properties and reinforced nanoindentation parameters (hardness and elastic modulus), which could further benefit their potential use in clinical applications. Additionally, both macro- and nano-mechanical properties were higher in films containing only gly (Group 1) compared to those containing both gly and PEG 1500 (Group 2). The incorporation of NHs into the formulations determined the hydrophilic character of the films, which is considered to be a key property in terms of maintaining adequate moisture in the wound. Furthermore, in vitro cytotoxicity assays showed that all the films were compatible with keratinocyte cultures.

CS/HC-NH-loaded films may have promising characteristics in terms of wound care applications, providing hydrophilic surfaces, adhesiveness, and toughness compared to CS/HC films. Future research should focus on exploring the in vitro and in vivo efficacy of NH-loaded films to clearly define their potential applications in biomedicine.

## Figures and Tables

**Table 1:** Composition of the blends prepared for film formation.

	Film	CS (% *w*/*v*)	HC (% *w*/*v*)	Span^®^ 85 (% *w*/*v*)	Plasticizer (g)		NHs (% *w*/*w*) *	
					gly	PEG 1500	HAL-CHX	HAL-hLF
Group 1	F1	1	2	-	0.6	-	-	-
	F1-10	1	2	0.05	0.6	-	5	5
	F1-20	1	2	0.05	0.6	-	10	10
Group 2	F2	1	2	-	0.6	0.6	-	-
	F2-10	1	2	0.05	0.6	0.6	5	5
	F2-20	1	2	0.05	0.6	0.6	10	10

* relative to the total mass of CS and HC.

**Table 2:** Thickness and weight of the films (mean values ± s.d; n = 5).

	Film	Thickness (μm)	Weight (mg)
Group 1	F1	128.39 ± 0.07	375.18 ± 0.01
	F1-10	179.00 ± 0.03	423.66 ± 0.00
	F1-20	228.90 ± 0.06	428.16 ± 0.01
Group 2	F2	152.31 ± 0.07	453.97 ± 0.01
	F2-10	185.29 ± 0.02	483.68 ± 0.02
	F2-20	271.88 ± 0.12	556.82 ± 0.04

**Table 3:** Macroscopic mechanical properties of the films (mean values ± s.d.; n = 5).

	Film	TS (MPa)	EB (%)
Group 1	F1	0.93 ^a^ ± 0.10	102.8 ^a’^ ± 6.4
	F1-10	0.66 ^b^ ± 0.14	124.5 ^b’^ ± 12.3
	F1-20	0.54 ^c^ ± 0.11	104.6 ^c’^ ± 9.5
Group 2	F2	0.62 ^d^ ± 0.12	84.0 ^d’^ ± 6.3
	F2-10	0.54 ^e^ ± 0.12	101.8 ^e’^ ± 12.4
	F2-20	0.42 ^f^ ± 0.07	93.3 ^f’^ ± 10.1

One-way ANOVA, post hoc Scheffé test (*p* < 0.05: a vs. b, c, d; d vs. f; a’ vs. b’, d’; b’ vs. c’; d’ vs. e’), e and f’: no significant.

**Table 4:** Nanomechanical properties of the films extracted using the AFM force spectroscopy measurements (mean values ± s.d; n = 30).

	Film	Stiffness (nN)	Young’s Modulus (nPa)	Adhesion Force (nN)
Group 1	F1	712.07 ± 16.35	81.82 ± 19.32	375.52 ± 96.82
	F1-10	701.71 ± 19.71	141.95 ± 34.55	900.03 ± 357.44
	F1-20	701.72 ± 24.42	46.02 ± 21.19	1878.83 ± 686.06
Group 2	F2	728.75 ± 31.04	145.23 ± 47.53	518.87 ± 174.28
	F2-10	738.76 ± 23.73	130.11 ± 42.29	2046.15 ± 860.68
	F2-20	683.81 ± 47.56	93.44 ± 47.56	1376.41 ± 739.92

**Table 5:** Nanomechanical properties of the films from nanoindentation measurements (mean values ± s.d; n = 8–16).

	Film	H (GPa)	E_r_ (GPa)
Group 1	F1	0.011 ^a^ ± 0.00	0.219 ^a’^ ± 0.01
	F1-10	0.017 ^b^ ± 0.00	0.341 ^b’^ ± 0.02
	F1-20	0.016 ^c^ ± 0.01	0.359 ^c’^ ± 0.09
Group 2	F2	0.006 ^d^ ± 0.00	0.125 ^d’^ ± 0.01
	F2-10	0.012 ^e^ ± 0.00	0.209 ^e’^ ± 0.02
	F2-20	0.018 ^f^ ± 0.01	0.321 ^f’^ ± 0.09

One-way ANOVA, post hoc Scheffé test (*p* < 0.05: a vs. b, c, d; b vs. e; d vs. e, f; e vs. f; a’ vs. b’, c’, d’; b’ vs. e’; c’ vs. f’; d’ vs. e’, f’; e’ vs. f’).

**Table 6:** Contact angles and surface free energy components of the films (mean values ± s.d; n = 6).

	Film	Contact Angle (θ, Degrees)			Surface Free Energy Components (mJ/m^2^)		
		H_2_O	Formamide	Diiodomethane	γ_S_^LW^	γ_S_^+^	γ_S_^-^
Group 1	F1	83.2 ± 6.3	67.9 ± 6.4	98.2 ± 8.2	9.35 ± 3.08	7.31 ± 0.70	6.42 ± 2.26
	F1-10	48.8 ± 8.7	76.1 ± 12.6	81.5 ± 2.5	16.74 ± 1.24	0.16 ± 0.71	73.60 ± 3.37
	F1-20	58.5 ± 5.5	74.0 ± 4.1	50.5 ± 6.3	33.98 ± 3.54	2.70 ± 0.25	52.30 ± 5.55
Group 2	F2	87.3 ± 7.2	91.3 ± 6.6	129.2 ± 9.5	1.71 ± 1.20	4.92 ± 0.24	17.01 ± 4.42
	F2-10	58.9 ± 3.8	73.7 ± 5.0	62.1 ± 7.0	27.36 ± 4.04	0.98 ± 0.02	50.24 ± 0.97
	F2-20	49.7 ± 2.9	57.5 ± 5.4	48.8 ± 5.5	34.93 ± 3.05	0.17 ± 0.11	46.65 ± 1.35

## Data Availability

Data is contained within the article.
